# The role of early social rearing, neurological, and genetic factors on individual differences in mutual eye gaze among captive chimpanzees

**DOI:** 10.1038/s41598-020-64051-y

**Published:** 2020-05-04

**Authors:** William D. Hopkins, Michele M. Mulholland, Lisa A. Reamer, Mary Catherine Mareno, Steven J. Schapiro

**Affiliations:** 1 Department of Comparative Medicine, The University of Texas MD Anderson Cancer Center, Michale E. Keeling Center for Comparative Medicine and Research, 650 Cool Water Drive, Bastrop, Texas 78602 USA; 2 Center for Behavioral Neuroscience, Georgia State University, 100 Piedmont Ave SE, Atlanta, Georgia 30303 USA; 3 Department of Experimental Medicine, University of Copenhagen, Blegdamsvej 3, 2200 Copenhagen N, Copenhagen, Denmark

**Keywords:** Cognitive neuroscience, Behavioural genetics

## Abstract

Mutual eye gaze plays an important role in primate social development and communication. In the current study, we examined the underlying experiential, genetic, and neuroanatomical basis of mutual eye gaze variation in adult captive chimpanzees. A multivariate analysis of variance revealed a significant rearing effect on bout length, with human-reared chimpanzees engaging in longer bouts of mutual gaze compared to mother-reared and wild-born individuals. Next, we utilized source-based morphometry (SBM) to examine gray matter covariation in magnetic resonance imaging scans and determine the relationship between the resulting gray matter covariation components and mutual eye gaze. One SBM component was negatively correlated with gaze duration (nucleus accumbens and anterior insular cortex), while two components were positively correlated with bout length (posterior cingulate cortex, inferior occipital cortex, middle temporal cortex, hippocampus, and the precentral sulcus). Finally, heritability analyses revealed mutual eye gaze to be modestly heritable and significant genetic correlations between bout length and two gray matter covariation components. This study reveals that non-genetic factors, and to a lesser extent, genetic factors appear to influence mutual eye gaze in adult chimpanzees, and is the first to report neuroanatomical correlates of mutual eye gaze variation in chimpanzees.

## Introduction

Visual communication through mutual eye gaze and gaze following play important roles in social development and nonverbal communication in human and nonhuman primates^1–5^. For instance, early in life, infants and their caregivers engage in mutual eye gaze and many have suggested that this early nonverbal communication has a facilitating effect in the formation of cognition, social bonding, and attachment^6–8^. Further, deficits in mutual eye gaze have been reported in clinical populations that are characterized as exhibiting atypical social, behavioral, and socio-communicative skills, such as schizophrenia and autism spectrum disorder^9–11^. Others have suggested that mutual eye gaze plays a role in the ways that individuals make inferences about what others can see and may think. Indeed, Grossmann^1^ hypothesized that, in humans, responses to mutual eye gaze and gaze following play the key functions of detecting the presence, inferring the content, and fostering collaboration with other minds.

Given the importance of visual communication in primate communicative signaling, researchers have published many comparative studies on the structure of the eye, as well as the function of gaze in communicative behavior in nonhuman primate taxa, from prosimians to great apes^12–20^. The collective findings suggest that information conveyed through mutual eye gaze and gaze following is widespread among primate species, and therefore, has a strong evolutionary foundation. However, our understanding of individual and phylogenetic variation in the neural and biological foundations of mutual eye gaze and gaze following is limited and relatively understudied^9,16,21–24^.

In the current study, one goal was to examine the independent and potential interactive effect of experiential and genetic factors on chimpanzee mutual eye gaze (MEG). Our motivation for this aspect of the study was two-fold. First, though there is widespread comparative interest in gaze-related behavior, such as mutual eye gaze and gaze following in nonhuman primates, there are few studies that have examined the potential role of genetic factors underlying individual variation in relatively large cohorts of individuals. Consistent with previous studies in nonhuman primates that utilize pedigree information from captive populations, we used the software program SOLAR to estimate heritability in the MEG measures^25–32^. Given the strong evolutionary foundations for MEG observed in primates, we hypothesized that MEG would be significantly heritable.

Second, in addition to estimating the contribution of genetic factors to MEG, we also sought to examine how experiential factors may contribute to individual variation in MEG. Specifically, in chimpanzees and other great apes, there is some evidence that early rearing experiences have a significant influence on measures of social cognition, including gaze following, and in responses to socio-communicative cues, such as gazing and manual pointing. In the majority of these studies, chimpanzees with extensive experience with humans, or enculturated apes as they are sometimes called, outperform apes raised in standard laboratory settings^20,33–37^. One interpretation of the enhanced performance of encultured apes is that they are more sensitive to or have learned to better respond to human socio-communicative cues than chimpanzees raised by, and with, their conspecifics. Because humans often serve as the experimenter in studies of social cognition in apes, the suggestion is that they perform better because they are better able to utilize early experiences with humans on certain tasks. To examine how different experiences may influence MEG, we evaluated the role of early social rearing on MEG variation by comparing chimpanzees reared by humans, to those raised by their biological mothers in captivity, or were born in the wild. Because the human-reared apes in the current study were more familiar with humans early in life, it was hypothesized that these chimpanzees would engage in mutual eye gaze more frequently, and for longer durations, than conspecific mother-reared or wild-born apes after controlling for their genetic relatedness.

A second goal of this study was to examine the neuroanatomical correlates of MEG in chimpanzees. MEG measures were correlated with gray matter covariation component scores derived from a source-based morphometry (SBM) analysis^38^ performed on magnetic resonance image scans obtained in the chimpanzee sample. Our research group has previously performed SBM analysis on scans from a sample of captive chimpanzees (the majority of which were subjects in the current study) and found between 19 and 24 spatial components, depending on the preprocessing methods^25^. Here, phenotypic measures of MEG were correlated with the weighted scores derived from each component to determine whether individual variation was associated with covariation scores for any of the components. Finally, to test whether any phenotypic associations we found between the MEG scores and the SBM components had a potential common genetic basis, we used quantitative genetics to test for genetic correlations in our chimpanzee sample.

## Results

### Reliability and the effect of sex and rearing on mutual eye gaze

Pearson’s product-moment correlations were performed to test for consistency in MEG responses across the four trials and these findings are shown in Table 1. We found significant positive associations among the four trials for mutual eye gaze frequency, duration, and bout length. Thus, the chimpanzee responses were consistent and repeatable across test trials. For this reason, we averaged the frequency, duration, and bout length measures of mutual eye gaze across all four trials and these three variables served as the outcome measures for all subsequent analyses. We also assessed inter-observer reliability by having two researchers collect simultaneous data, with their heads side-by-side, from a subset of chimpanzees (NCCC = 40; YNPRC = 11). This is by no means a perfect assessment of reliability, but both frequency (*r* = 0.89–0.92) and duration (*r* = 0.93–0.96) were highly positively correlated between observers.

**Table 1:** Pearson Product-Moment Correlation Coefficients between Mutual Eye Gaze Frequency, Duration and Bout Length Across the 4 Trials.

Frequency			
	Trial 2	Trial 3	Trial 4
Trial 1	0.354	0.283	0.368
Trial 2	—	0.417	0.344
Trial 3	—	0.403	
**Duration**			
	Trial 2	Trial 3	Trial 4
Trial 1	0.409	0.394	0.395
Trial 2	—	0.454	0.552
Trial 3	—	0.542	
**Bout Length**			
	Trial 2	Trial 3	Trial 4
Trial 1	0.303	0.351	0.290
Trial 2	—	0.366	0.576
Trial 3	—	0.428	

All correlation coefficients were significant at *p* < 0.001.

**Figure 1:**
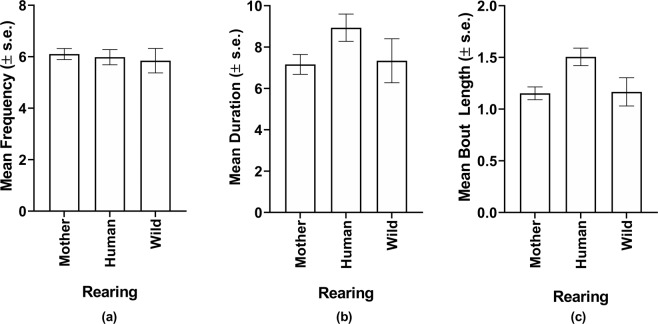
Mean and standard errors bars for mutual eye gaze (**a**) frequency (**b**) duration and (**c**) bout length in the mother-reared, human-reared, and wild-born chimpanzees.

We next considered the effect of sex and rearing on each average mutual eye gaze outcome measure. For this analysis, a multivariate analysis of covariance was initially performed, with the mean frequency, duration, and bout length in mutual eye gaze serving as the dependent measures, age as a covariate, and sex and rearing history as the fixed factors. An overall significant main effect for rearing was found [*F*(6, 422) = 2.595, *p* = 0.018]. The subsequent univariate *F*-tests revealed significant rearing effects on bout length [*F*(2, 211) = 6.5f15, *p* = 0.002], and a borderline effect for duration [*F*(2, 211) = 2.689, *p* = 0.070]. There was no significant effect for frequency [*F*(2, 211) = 0.124, *p* = 0.883]. The mean mutual eye gaze frequency, duration, and bout length are shown in Fig. 1. For bout length and duration, post-hoc analyses using Tukey’s Honestly Significant Difference tests, indicated that human-reared chimpanzees had longer bout durations than mother-reared and wild-born individuals, but these latter two groups did not differ significantly from each other. Human-reared chimpanzees had longer total durations than both mother-reared and wild-born chimpanzees, but this difference was only significant for wild-born individuals. There was no significant difference in mutual eye gaze across the three rearing conditions.

### Phenotypic associations between mutual eye gaze and the SBM correlates of mutual eye gaze

**Figure 2:**
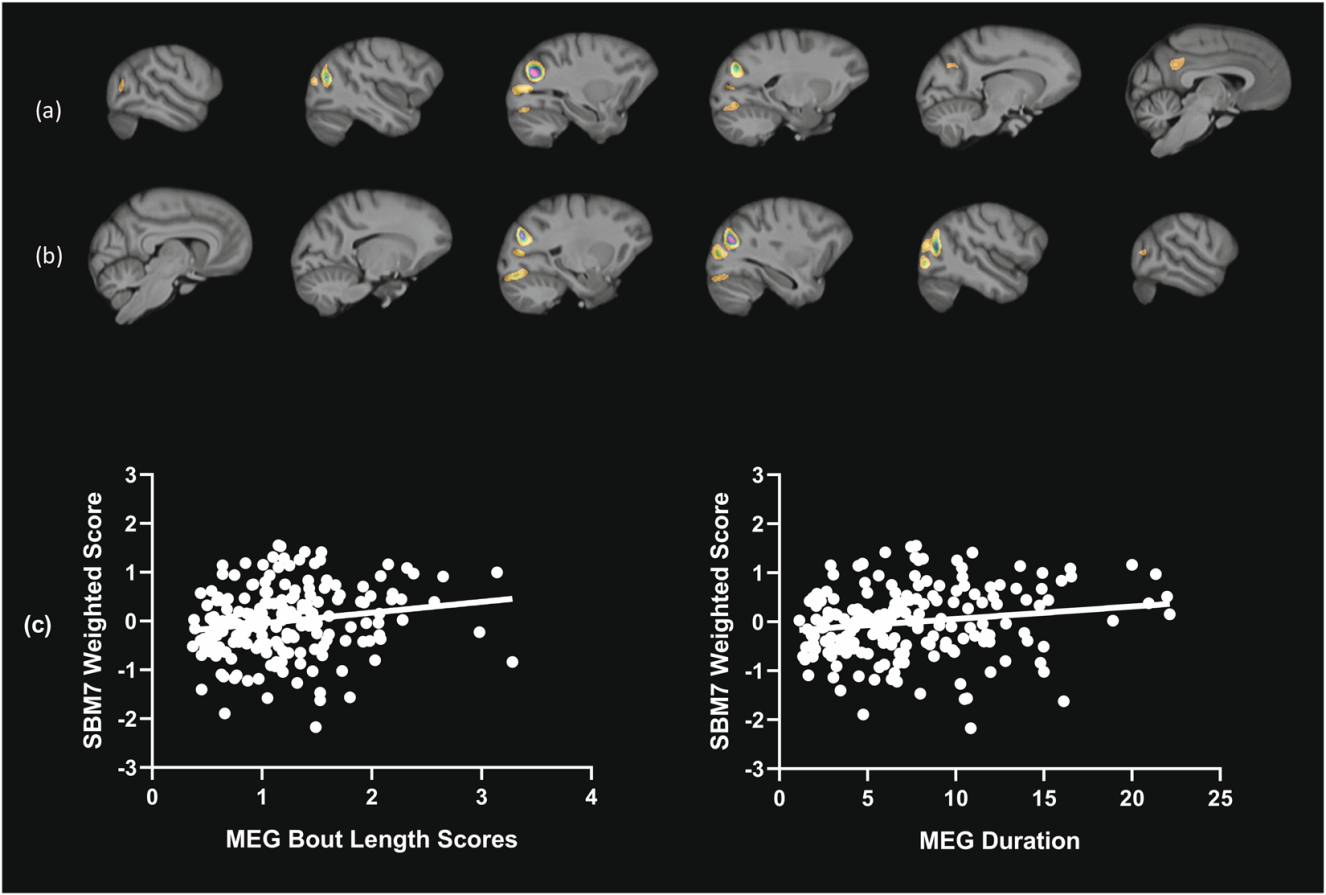
(**a,b**) Serial sagittal views of visual cortical and the posterior cingulate regions that comprise SBM component 7, and (**c**) scatterplots of the association between SBM7 scores and mean bout length and overall duration. *Note: Weighted scores are adjusted for covariates*.

We next tested for associations between the different measures of mutual eye gaze and the weighted score for each SBM component. Partial correlation coefficients were performed between the mutual eye gaze frequency, duration, and bout length measures and the subject’s weighted score for each of the 18 SBM components, with scanner magnet, sex, age, and rearing history as covariates. We found no significant associations between the frequency in mutual eye gaze and any of the SBM components. For the duration in mutual eye gaze, a significant positive association was found with SBM component 7 (*r* = 0.163, *p* = 0.026) and a negative association with SBM component 18 (*r* = −0.145, *p* = 0.049). Lastly, for mutual eye gaze bout length, significant positive associations were found with SBM components 7 and 10 (*r* = 0.174, *p* = 0.018 and *r* = 0.212, *p* = 0.004, respectively), and a significant negative association with component 18 (*r* = −0.160, *p* = 0.030). Scatterplots showing the association between mutual eye gaze duration and bout length, and the weighted scores for SBM components 7, 10 and 18 are shown in Figs. 2 through 4. SBM component 7 was comprised of the posterior cingulate cortex (bilateral) and inferior occipital cortex (bilateral). SBM component 10 was made up of bilateral anterior and posterior middle temporal cortex, tail of the hippocampus and the ventral portion of the left inferior precentral sulcus. SBM component 18 was comprised of two main regions, including the inferior basal forebrain, which included the nucleus accumbens and anterior insular cortex.

**Figure 3:**
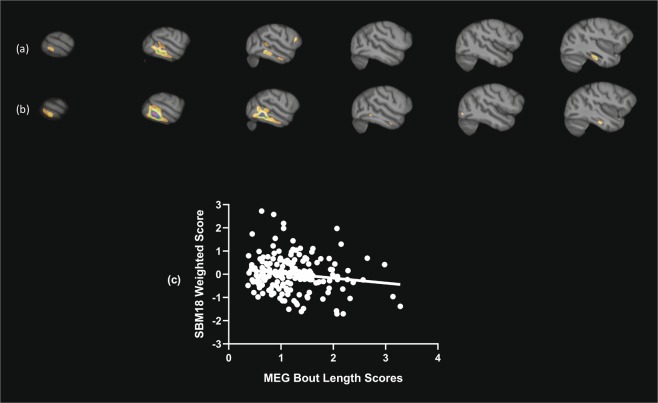
(**a,b**) Serial sagittal views of middle and inferior temporal lobe and hippocampal regions that comprise SBM component 10, and (**c**) scatterplot of the association between mean bout length and SBM10 scores. *Note: Weighted scores are adjusted for covariates*.

**Figure 4:**
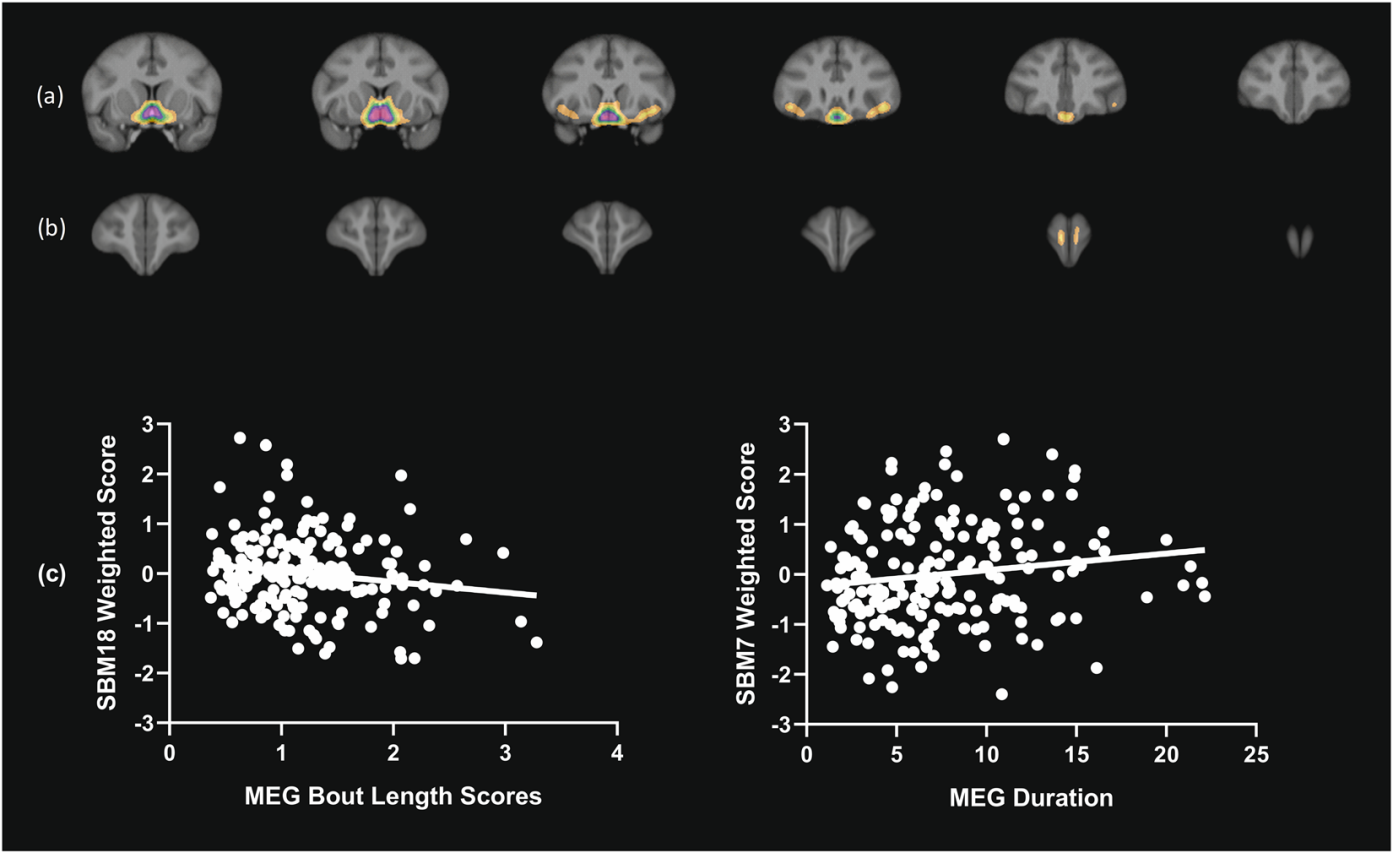
(**a,b**) Serial coronal views of basal forebrain, anterior insula and frontal pole regions that comprise SBM component 18, and (**c**) scatterplots of the association between SBM18 scores, and mean bout length and overall duration. *Note: Weighted scores are adjusted for covariates*.

### Heritability and genetic correlations between mutual eye gaze and SBM components

In the next set of analyses, we estimated heritability for the measures of MEG and those SBM components (7, 10 and 18) that were found to be phenotypically associated with them. Table 2 shows the heritability estimates and contributions of the covariates on each MEG phenotype and SBM components 7, 10, and 18. As can be seen, significant heritability was found for frequency in mutual eye gaze, while borderline significant heritability was found for duration and mean bout length. With respect to the SBM components, all three were significantly heritable. Not surprisingly, scanner magnet also accounted for a significant proportion of variance, as did to a lesser extent, sex and age. We next tested for genetic correlations between the mutual eye gaze mean bout length data and the weighted scores for SBM components 7, 10 and 18, respectively. We found a significant genetic correlation between mutual eye gaze mean bout length and SBM component 7 (rhoG = 0.896, SE = 0.707, p = 0.007) and a borderline significant correlation with component 10 (rhoG = 0.872, SE = 1.14, p = 0.073). There was no significant genetic correlation between mutual eye gaze bout length and SBM component 18.

**Table 2:** Heritability of MEG Measures and SBM components.

Phenotype	h^2^	s.e.	p	Covariates	Variance
**Mutual Gaze**					
Frequency	0.381	0.167	0.006	Rearing	0.034
Duration	0.183	0.148	0.081	None	
Bout Length	0.186	0.129	0.050	None	
**SBM Components**					
7	0.765	0.134	0.000001	Sex, Age, Scanner	0.344
10	0.907	0.109	0.000001	Sex, Age, Scanner	0.192
18	0.391	0.146	0.001	Age, Scanner	0.312

## Discussion

There were five main findings in the current study. First, we found consistent MEG responding across four test trials separated by at least one day. Thus, as a measure of individual variation, MEG seems to be a reliable measure. Second, we found that early social rearing experiences had a significant impact on mutual eye gaze bout length. Third, measures of MEG were modestly heritable in our sample of chimpanzees. Fourth, we found significant neuroanatomical associations with individual variation in MEG bout length and duration, but not frequency. Finally, we found significant genetic correlations between MEG bout length and two of the three gray matter covariation components.

Regarding rearing history, we found that chimpanzees reared by humans in a nursery setting engaged in mutual eye gaze for longer bout lengths compared to mother-reared and wild-born chimpanzees. There was also a borderline, but non-significant, effect of overall duration, with nursery-reared chimpanzees having longer overall duration of mutual eye gaze. There was no difference, however, in the frequency of mutual eye gaze across these rearing histories. These findings were consistent with our hypothesis, and suggest that early social rearing experiences can shape MEG and, perhaps, tune them toward social agents early in life as has been reported in humans^39^. It might be argued that the nursery-reared chimpanzees simply had more exposure to humans than mother-reared or wild-born apes, and this explains the results; however, the wild-born apes were older individuals that were brought to captivity before 1974, when the importation of wild chimpanzees ended. Thus, these apes have spent as much time around, and interacting with, humans as many, if not all, of the nursery-reared apes. If it was simply a matter of exposure to humans accounting for the rearing effect, then the data from the wild-born apes should be more similar to the nursery- than mother-reared apes. We believe what accounts for the increased duration and bout length in the nursery-reared chimpanzees is that they received this eye contact early in life facilitating their MEG responding as adults.

The findings showing increased MEG duration and bout length in nursery-reared compared to mother-reared chimpanzees are relevant to other existing literatures in, at least, two important ways. First, previous studies have shown that nursery-reared chimpanzees, particularly those with intensive interactions with humans, perform significantly better (or more similar to humans) on tasks that measure dimensions of social cognition, such as gaze following, joint attention, understanding of intentions, and the use of attention-getting behaviors compared to mother-reared apes^33,34,40^. In light of the fact that many of these cognitive tasks involve the chimpanzees monitoring of the experimenter’s (a human) visual attention, it seems reasonable to suggest that early exposure to human mutual eye gaze may account for performance on these, and other, social cognition tasks. Second, there is some evidence that nursery-reared chimpanzees have a difficult time integrating in social groups of chimpanzees as juveniles, adolescents or adults, particularly if the existing group is comprised of a disproportionately large number of mother-reared individuals^41–43^. One potential explanation for the difficulty of nursery-reared chimpanzees to integrate into larger chimpanzee social groups comprised of mother-reared individuals is that they have not learned the appropriate species-specific functional uses of, and responses to, different aspects of mutual eye gaze in social contexts. This is not to suggest that they cannot or do not eventually learn them, but their acquisition of these skills is delayed and programmed differently, and therefore, requires additional experiences.

One limitation of this study was the fact we used a human experimenter to study MEG rather than trying to quantify these measures during intraspecific interactions between chimpanzees^44^. Thus, the ecological validity of these findings might be called into question. Measuring mutual eye gaze with a human observer, while likely less representative of natural gaze behaviors in the wild, is reliable and certainly more ecologically valid than using video or pictorial stimuli in studies involving eye tracking and related methodologies. Eye gaze behaviors and responses to faces are of great interest in both human and nonhuman primate research, most of which involves automated presentation of two-dimensional stimuli (videos or pictures)^19,45–50^. While eye tracking and related studies clearly show that features of the face are a focal point of interest, it is not necessarily the case that nonhuman primates view them as representational social stimuli^51^. Indeed, Rossion and Taubert^48^ have suggested that nonhuman primates do not see images on the screen as social stimuli, even though they can discriminate between faces and non-faces using these methods. In short, we believe that the approach used in this study is ecologically valid, though this could be improved with additional studies. While more difficult to collect, the frequency and duration of mutual eye gaze with a conspecific should be directly compared to those with a human observer.

MEG also showed moderate to low heritability, suggesting that genetic factors likely play only a small role in explaining individual variation in chimpanzees, a finding that did not support our hypothesis. We were somewhat surprised by this result, given (1) the role mutual eye gaze and gaze following play in social communication in chimpanzees and other primates and (2) our previous findings demonstrating heritability in gaze following and related social cognitive processes in chimpanzees^52,53^. One possibility is that the variability in MEG responses, particularly the average bout length, was relatively low, constraining our ability to detect stronger heritability (i.e., the range of responses was too small). Indeed, in humans, bonobos, chimpanzees, and monkeys, eye tracking studies of conspecific faces clearly show that the eyes are a region of interest (though there are species differences in amount of time spent looking at the eyes versus other regions)^19,50^. Thus, the eyes are a very potent visual stimulus in primates and automatic responses to facial stimuli likely attenuate individual variation in MEG. Alternatively, it may be that social learning and experiential factors play a far greater role in shaping the function and use of mutual eye gaze in social communication in primates than genetic factors, an interpretation partially supported by the rearing effects reported in this study.

We also found phenotypic associations between MEG bout length and three SBM components (7, 10 and 18) and genetic correlations for two components (7 and 10). Recall that SBM components 7 and 10 were comprised of visual cortex and portions of the inferior and middle temporal cortex. The regions within SBM components 7 and 10 are part of the visual processing stream, particularly the ventral pathway, and therefore, it is not surprising that these areas are both phenotypically and genetically associated with MEG. The association between SBM component 18 and MEG bout length is more difficult to explain, yet interesting for at least two reasons. First, it has been previously reported that mother- and nursery-reared chimpanzees differ on gray matter covariation in the basal forebrain^54^, and this region entirely overlaps with those described for component 18 in this study. Recall that nursery- and mother-reared chimpanzees differed in their MEG bout length, and that MEG bout length is associated with covariation in the basal forebrain region, suggesting that rearing experiences may mediate the association between MEG and gray matter covariation within the basal forebrain. Second, regions within the basal forebrain, including the caudate and nucleus accumbens, are rich in dopamine neurons, and some have suggested that they play an important role in the reward circuit of the brain^55^. It has also been suggested that mutual eye gaze activates oxytocin release during social interactions, which in turn, stimulates the release of dopamine^56–58^. This results in a feedback loop between oxytocin and dopamine that facilitates the reward value of mutual eye gaze during social interactions^59^. Therefore, the association between MEG bout length and SBM component 18 reported here, may reflect individual variation in the reward value of mutual eye gaze for the chimpanzees. This interpretation is somewhat speculative because we have no direct evidence for their association, but warrants further investigation.

In summary, the collective findings of this study reveal that non-genetic factors, and to a lesser extent, genetic factors appear to influence mutual eye gaze in adult chimpanzees. Additionally, individual variation in mutual eye gaze bout length is associated with gray matter covariation in the visual cortex, inferior and middle temporal cortex, and the basal forebrain. These findings are the first systematic report of neuroanatomical correlates of individual variation in chimpanzee mutual eye gaze; however, more studies are needed to further identify the neurogenomic mechanisms and consequences that underlie variation in mutual eye gaze. Continued studies in chimpanzees and other primates will further advance our understanding of the neurochemical, psychological, evolutionary, and potential clinical relevance of mutual eye gaze.

## Methods

### Subjects

For the behavioral studies, there were 219 adult chimpanzees (*Pan troglodytes*; 11–59 years old; 138 females, 81 males) from two facilities: the Yerkes National Primate Research Center (YNPRC, N = 69) and the National Center for Chimpanzee Care (NCCC, N = 150) of The University of Texas MD Anderson Cancer Center. All chimpanzees were socially housed with 24-hour access to indoor/outdoor enclosures (except during cleaning) with bedding, climbing structures, and daily environmental enrichment. Chimpanzees were fed a commercially available primate diet and fresh produce, with daily foraging opportunities and *ad libitum* access to water.

Of the 219 subjects, we included 125 mother-reared, 52 nursery-reared, and 42 wild-born apes. As we have previously defined^12,60^, nursery-reared chimpanzees were separated from their mother (between 0–30 days old) due to unresponsive care, injury, or illness. These infants were hand-reared and fed human infant formula (not supplemented with DHA as far as we know) until they could sufficiently care for themselves. They were placed with same-age peers until three years old when they were integrated into larger mixed-age social groups (including adults and sub-adults)^12,60^. Mother-reared chimpanzees were reared by their mother in their natal groups (4–20 chimpanzees) for at least the first 2.5 years of life. Wild-born apes were brought to the United States prior to the 1974 CITES importation ban. Of the 219 chimpanzees for which behavioral data were available, we also had structural brain scans available for 191 individuals (79 males, 112 female; 104 mother-reared, 54 nursery-reared, and 33 wild-born). All work was approved by the Institutional Animal Care and Use Committees at both NCCC and YNPRC and adhered to the ethical guidelines of National Institutes of Health.

### Mutual eye gaze

A familiar experimenter (over 8 years of experience with the chimpanzees) tested each individual during four 1-minute test trials that were separated by at least one day. Each trial began with the experimenter greeting the subject and giving the subject (and any present group members) a small piece of food (i.e., a peanut or grape). The experimenter then placed the food out of sight of the apes, and sat or stood (depending on the subject’s position) approximately 2–3 feet from the subject’s home cage (close enough to feel confident knowing when eye contact was achieved). A trial did not begin until the subject appeared settled and focused on the experimenter with outside activity and noise at a minimum. To initiate a trial, the experimenter made direct eye contact with the subject. For the subsequent 60 seconds, the total amount of time and frequency of mutual eye gaze events were recorded with a stopwatch by the experimenter. Throughout the 1-minute trial, the experimenter actively sought to maintain eye contact with the subject, including calling its name, if for some reason it turned away from the experimenter. Though rare in occurrence, trials were terminated if the subject left the area and went out of view from the experimenter, or if an event (i.e., alarm barking, fighting in the group) caused the subject’s attention to be significantly diverted. For example, chimpanzees at NCCC were only distracted and required re-testing for 6 trials. Subjects were tested in their social groups and no attempt was made to isolate them for testing. For each trial, the number of mutual eye gaze events and the total duration in mutual eye gaze were recorded and served as dependent measures in subsequent analyses. Additionally, average bout length of mutual eye gaze was computed for each trial by dividing the total duration of MEG by the frequency of MEG events.

### MRI acquisition and post-image processing

We used magnetic resonance image (MRI) scans that were acquired during one of the chimpanzee’s past annual physical examinations (as previously reported^25,61–63^). Briefly, the animals were first sedated, then subsequently anesthetized, before being transported to a mobile MRI facility. For safety purposes, chimpanzees were briefly housed alone for 2–24 hours while recovering from anesthesia before being reunited with their social group. All procedures were approved by the Institutional Animal Care and Use Committees at YNPRC and NCCC, and also followed the guidelines of the Institute of Medicine on the use of chimpanzees in research. For this study, 77 YNPRC chimpanzees were scanned using a 3.0 Tesla scanner (Siemens Trio, Siemens Medical Solutions USA, Inc., Malvern, Pennsylvania, USA), while the remaining 149 chimpanzees (11 from YNPRC, 138 from NCCC) were scanned using a 1.5T GE echo-speed Horizon LX MR scanner (GE Medical Systems, Milwaukee, Wisconsin, USA).

Following acquisition, we used a Macintosh computer to process all of the MRI scans using recently published procedures^63^. We completed all processing twice (for the 1.5T and 3T scans separately). Briefly, we converted raw DICOM files into NiFTI format using 3D Slicer 4 (www.3Dslicer.org)^64,65^, then used the brain extraction function in FSL for skull stripping^66,67^. Subsequently, we bias corrected and denoised each image using 3D Slicer^68–71^ and the DWI Denoising Package for MATLAB (R2015b; Mathworks, Natick, Massachusetts, USA)^72,73^. We then resampled the scans (at 0.625 mm), aligned each on the AC-PC axis, and saved them in radiological space (all subjects had a directional marker placed before imaging) using ANALYZE 11.0 (AnalyzeDirect, Overland Park, Kansas, USA). Finally, we registered each scan to a chimpanzee template brain^61^ using a 12-parameter affine linear registration in FSL^74,75^.

The AC-PC-aligned and affine-registered brains were then processed through the FSL-VBM pipeline (http://fsl.fmrib.ox.ac.uk/fsl/fslwiki/FSLVBM) and subsequently reregistered to the chimpanzee template brain, using a 6-parameter rigid body linear registration in FSL (FLIRT)^61,74,75^, ensuring that the 1.5T and 3T scans had the same orientation and voxel dimensions (see detailed processing methods in^63^).

### SBM analysis

The SBM analysis was performed with the software program available in the toolbox GIFT (http://icatb.sourceforge.net) on the smoothed, modulated gray matter volumes for the 191 chimpanzees for which MRI and MEG data were available. The individual chimpanzee’s smoothed gray matter volumes were imported into the program and subjected to an independent component analysis using the default parameters. For this set of MRI scans, the SBM produced 18 gray matter components. SBM then computes a weighted score for each subject that reflects their contribution to the creation of each gray matter covariation component. These weighted scores for the 18 components were the outcome measures of interest for subsequent phenotypic and heritability analyses.

### Heritability analyses

We used the software program SOLAR^76^ to determine the heritability of each MEG measure (see^25^ for detailed methods). Researchers have used SOLAR extensively with nonhuman primates for a variety of behavioral and brain phenotypes see^26,28,30,31,52,53,77–80^. We used SOLAR to estimate heritability based on the chimpanzee pedigree, and to determine whether the three MEG measures were significantly heritable. We then used SOLAR to run genetic correlations (RhoG) to determine the extent to which common genetic mechanisms explained both the MEG measures and the correlated SBM component scores. We included sex, age, rearing history, and scanner magnet (when appropriate) as covariates for all genetic analyses.

## Data Availability

All MRI scans and datasets analyzed in the current study will be available through the National Chimpanzee Brain Resource data repository (www.chimpanzeebrain.org).
